# Assessing cell-specific effects of genetic variations using tRNA microarrays

**DOI:** 10.1186/s12864-019-5864-1

**Published:** 2019-07-16

**Authors:** Christine Polte, Daniel Wedemeyer, Kathryn E. Oliver, Johannes Wagner, Marcel J. C. Bijvelds, John Mahoney, Hugo R. de Jonge, Eric J. Sorscher, Zoya Ignatova

**Affiliations:** 1 Biochemistry and Molecular Biology, Department of Chemistry, University of Hamburg, 20146 Hamburg, Germany; 2 Emory University School of Medicine, Atlanta, GA 30322 USA; 3 Children’s Healthcare of Atlanta, Atlanta, GA 30322 USA; 4 Gastroenterology and Hepatology Erasmus MC University Medical Center, Rotterdam, The Netherlands; 5 Cystic Fibrosis Foundation CFFT Lab, Lexington, MA 02421 USA

**Keywords:** Nucleotide variants, Synonymous single nucleotide polymorphisms, tRNA, Protein translation, Cystic fibrosis

## Abstract

**Background:**

By definition, effect of synonymous single-nucleotide variants (SNVs) on protein folding and function are neutral, as they alter the codon and not the encoded amino acid. Recent examples indicate tissue-specific and transfer RNA (tRNA)-dependent effects of some genetic variations arguing against neutrality of synonymous SNVs for protein biogenesis.

**Results:**

We performed systematic analysis of tRNA abunandance across in various models used in cystic fibrosis (CF) research and drug development, including Fischer rat thyroid (FRT) cells, patient-derived primary human bronchial epithelia (HBE) from lung biopsies, primary human nasal epithelia (HNE) from nasal curettage, intestinal organoids, and airway progenitor-directed differentiation of human induced pluripotent stem cells (iPSCs). These were compared to an immortalized CF bronchial cell model (CFBE41o^−^) and two widely used laboratory cell lines, HeLa and HEK293. We discovered that specific synonymous SNVs exhibited differential effects which correlated with variable concentrations of cognate tRNAs.

**Conclusions:**

Our results highlight ways in which the presence of synonymous SNVs may alter local kinetics of mRNA translation; and thus, impact protein biogenesis and function. This effect is likely to influence results from mechansistic analysis and/or drug screeining efforts, and establishes importance of cereful model system selection based on genetic variation profile.

**Electronic supplementary material:**

The online version of this article (10.1186/s12864-019-5864-1) contains supplementary material, which is available to authorized users.

## Background

Genetic variations are the source of evolutionary diversity and are grouped into three general categories: deleterious, neutral and beneficial. Fitness landscapes, both at single-protein and whole-organism levels, are generally used to depict phenotypic manifestation of genotypes [1–3]. Predictive selection of variants with physiological effect is limited by the paucity of the quantitative systematic analysis of cellular contributors to variation in both tissues and cell lines. However, systematic assessment of the effect of genetic variations on the whole proteome level is laborious, and only a small set of proteins has been extensively examined to date (predominantly addressing effects of single nucleotide variants, SNVs) [4–9]. The outcome of a genetic change largely depends on overall genetic background [1, 10]. Interactions between genes and epistatic effects of intra- or inter-genic variations [11–20], together with quantitative differences in the components of central cellular processes (e.g. transcription, translation) contribute substantially to phenotypic heterogeneity. Furthermore, recent examples suggest that tissue-specific effects of certain forms of genetic variation are linked to components that comprise cellular translation machinery [21–23].

**Fig. 1:**
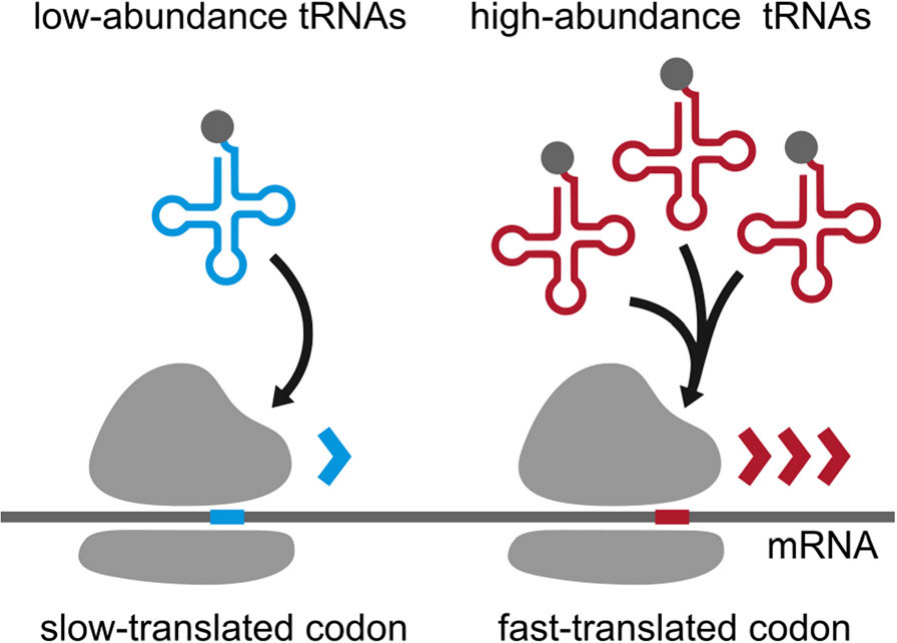
General model of mRNA translation. The rate of translation for each codon depends on the collision of tRNAs with the ribosome (gray) and is proportional to cellular concentration of the cognate tRNA isoacceptor [75]. Codons pairing to low-abundance tRNAs (blue) exhibit slower velocity than codons pairing to high-abundance tRNAs (red), which are translated more rapidly

Translation is a central process at the crossroads between genome and proteome. Large proportions of cellular resources are dedicated to this essential function: 35–45% of the genome is assigned to proteins of the translation apparatus, and ~ 30–50% of energy production with the cell is consumed by translation machinery [24]. Transfer RNAs (tRNAs) convert the nucleotide chemistry (mRNA) into a peptide alphabet (protein). ‘Ready-to-translate’ tRNA repertoires can adapt according to cellular physiology and are controlled by several transcriptional and posttranscriptional regulatory processes [25–27]. tRNAs have co-evolved to serve the degenerate DNA code, and many synonymous codons have a specific tRNA isoacceptor (i.e., a distinct tRNA carrying the same amino acid but with variation in the anticodon and tRNA body sequence). On the other hand, through modifications in the anticodon, some isoacceptors serve more than one synonymous codon. Cellular concentrations of tRNA isoacceptors vary greatly and shape behavior of synonymous codons, which in turn, has a profound effect on translation kinetics and accuracy (Fig. 1), as well as protein expression level, folding and activity [25–27]. Emerging knowledge regarding the ways tRNAs read synonymous codons has altered the view of synonymous SNVs, which have been historically considered inconsequential for protein folding and function as they change the codon but not the encoded amino acid. For example, a major determinant of elongation speed for a codon is the concentration of its cognate tRNA [28] and the ratio of cognate to near-cognate tRNA [29]. As a corollary of this view, the effect of synonymous SNVs on translation kinetics can be estimated from tRNA abundance. Here, we perform systematic global quantification of the tRNAomes within various human cell lines and model systems used in the study of cystic fibrosis (CF), and frame the effect of synonymous SNVs (sSNVs) in the context of cellular translation resources that directly link a particular SNV to cellular physiology (e.g. protein function). Rationale for working with CF cell models stems from the fact that the gene responsible for this disease – the cystic fibrosis transmembrane conductance regulator (*CFTR*) – encodes an abundance of well-classified SNVs (https://cftr2.org/) and synonymous nucleotide polymorphisms (SNPs, defined as SNVs with prevalence higher than 1% in the population [30]). Furthermore, theratype of disease-causing mutations in *CFTR*, along with other effects of newly developed modulator compounds, can be markedly non-uniform in different cell lines and disease models. Although tRNAs exhibit a similar global pattern of abundance among the tested models, specific isoacceptors oppose this trend; i.e., synonymous SNVs at CFTR codons read by those tRNAs would result in significant variation in different models. This interpretation is not restricted to *CFTR* and should also be useful for more global predictions regarding genotype-phenotype consequences attributable to synonymous SNVs.

## Results

### Rationale for model choice in tRNAome comparisons

CFTR functions as an epithelial anion channel and resides in the apical membranes of multiple tissues, including airways, hepatic and pancreatic ducts, as well as pancreatic acini and sweat ducts. In addition to the most prevalent cause of disease – deletion of three nucleotides (F508del) – more than 2000 CF-associated variants and SNPs have been identified in *CFTR*, which may impact both clinical severity and penetrance [31]. The FDA recently approved treatment of F508del-CFTR homozygous patients with combination drugs such as Orkambi™ and Symdeko™, which contain both a CFTR ion channel gating potentiator, ivacaftor (VX-770), and protein misfolding corrector compound, lumacaftor (VX-809) or tezacaftor (VX-661). Clinical testing of these agents has revealed considerable heterogeneity in patient response [32] that may reflect a number of factors, including influence of intragenic modifiers or specific differences in abundance of factors that govern drug effectiveness within cells and tissues. The number of compounds that influence specific CFTR mutations are rapidly increasing and may exhibit significant differences in effect depending on the model system being tested [33]. Our earlier studies of a synonymous SNP in CFTR (c.2562 T > G) revealed strong tissue-specific effects due to differences within tRNA households among distinct human tissues [22]. Thus, we hypothesized that variations in tRNA sets may alter CFTR translation profiles, and consequently, the effect of synonymous SNVs/SNPs in each system.

We determined tRNAomes from two CF patient-derived primary human bronchial epithelia (HBE) samples harvested at the time of lung biopsy and compared these to various models used in CF research for CFTR mechanistic analysis and/or drug screening. These model systems included intestinal organoids, Fischer rat thyroid (FRT) epithelia, an immortalized CF bronchial cell line (CFBE41o^−^), human nasal epithelia (HNE) from rhinal scrapings, and airway progenitor cells directed towards differentiation from human induced pluripotent stem cells (iPSCs). We also evaluated tRNAomes from two laboratory cell lines, HeLa and HEK293, which are commonly used in CF research for addressing molecular features of CFTR mutations during protein biogenesis.

The selection of these model systems was based on discussions resulting from an international Cystic Fibrosis Foundation workshop centered on CFTR theratyping with applications to both common and rare CFTR variants [34]. Patient-derived rectal organoids have become increasingly utilized for CF diagnostics, high-throughput drug screening (in 384-well format), patient-specific responsiveness/personalized theratyping, and testing FDA/EMA-approved drugs (e.g. Orkambi™) for effect(s) on rare CF mutations [34–36]. FRT cells have been extensively used in compound library screens by Vertex Pharmaceuticals, leading to discovery of clinically approved CFTR modulators such as ivacaftor, lumacaftor, tezacaftor, and a series of next-generation agents. Theratype protocols in the FRT model have been recognized by the FDA as potentially relevant to drug label expansion for individuals with unusual, ultra-rare, or even private SNVs in *CFTR*. Primary HNE cells are particularly useful for optimizing modulator choices among individual CF subjects with forms of the disease less suitable for definitive Phase III clinical trials [37]. The developments of new techniques to derive functional airway cell models from iPSCs using CF-patients’ somatic cells holds promise for in vitro modeling of CFTR theratype analysis, drug screening, and future cell-based regenerative therapies [38–40]. In the present study, we therefore tested iPSCs at: (1) day 0, following fibroblast depletion and at primitive streak induction, (2) day 5 upon dissociation of embryoid bodies, (3) day 15, upon lung progenitor induction, and (5) day 21, as expanded lung progenitors [41, 42]. We also included the CFBE41o^−^ cell line for comparison, since this model represents one of the most widely used immortalized cell models in basic CF research [43].

### Global tRNA abundance pattern is similar among models but differs for a specific population of codons

**Fig. 2:**
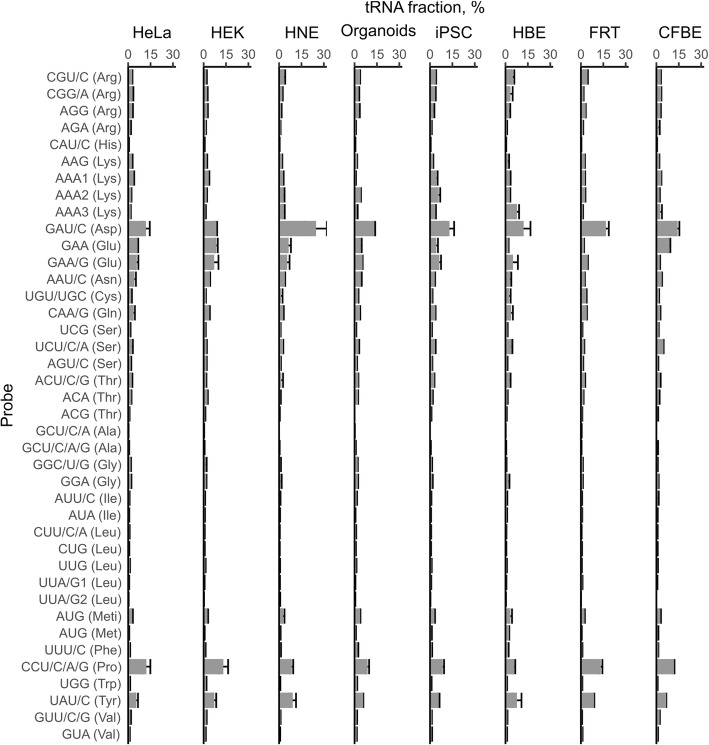
tRNAs exhibit similar concentration patterns among tested models. Values from comparative tRNA microarrays were converted into absolute concentrations using comparative arrays versus HeLa, and represented for each codon as a fraction of total tRNA. Data are presented as a representative array ± SD between biological replicates (*n* = 4 for HEK293; *n* = 3 for CFBE41o^−^, FRT and HeLa; *n* = 2 for organoids, HNE and HBE; for iPSCs (zero time point) *n* = 12 of the array blocks). tRNA isoacceptors are depicted with their cognate codon and the corresponding amino acid; Meti, initiator tRNA^Met^. The tRNAomes of HeLa and CBFE41o^−^ cells are from [22]

tRNAome profiles were obtained for each model system using tRNA-tailored microarrays [44] with 40 tDNA probes covering the full-length sequence of 49 nuclear-encoded cytoplasmic tRNAs as described previously [44, 45]. tRNA isoacceptors with a sequence difference of more than *8* nucleotides can be unambiguously determined in this fashion [46]. For example, specific isoacceptors pairing to proline codons cannot be unambiguously distinguished because they have fewer than 8 nt difference; while for other codons (e.g. AAA), more than one tRNA with variations in sequence encoding the tRNA body is detectable (Fig. 2). Notably, arrays showed a strong reproducibility between biological replicates (Additional file 1 Figure S1a-e). Distribution of overall tRNA abundancies was similar among the models – e.g. acceptors reading GAU/C (Asp) and UAU/C (Tyr) codons were highly expressed – whereas isoacceptors pairing to leucine codons exhibited lower abundance in all models evaluated (Fig. 2). Globally, tRNA fractions correlated among the tested models (Additional file 1 Figure S2a), but for some tRNA isoacceptor families (i.e. a family comprises all tRNA isoacceptors carrying the same amino acid), the proportion among the isoacceptors varied (Fig. 2). For example, in all cells within the Thr-tRNA family – except HEK293 – the isoacceptor with the highest concentration reads ACU/C/G, whereas the lowest one pairs to the ACG-codon. In HEK293, the tRNA reading ACA (Thr) was the most highly expressed within this family (Fig. 2). tRNAs^Glu^ and tRNAs^Arg^ displayed larger fluctuations in the proportion between isoacceptors within different model systems.

Over the time course of iPSC differentiation to an airway organoid phenotype, overall abundance and distribution of tRNAs remained relatively constant (Additional file 1 Figure S2b). Fractions of high- or low-abundance tRNAs mildly decreased or increased, respectively (Additional file 1 Figure S2b), although those changes did not reach statistical significance. Organoids and FRT cells exhibited the lowest correlation to the other models (Additional file 1 Figure S2a). On a global scale, the three model cell lines, HEK293, HeLa and CFBE41o^−^, were most similar to each other (Additional file 1 Figure S2a), with HEK293 cells being the most divergent (Fig. 2). It should be noted that modifications of these cells for propagation in laboratory conditions might alter their tissue-specific expression, i.e. deviation from the ancestral tissue may be difficult to assess. In addition, karyotypes of both HeLa and HEK293 are largely divergent from a human cell line, due to viral immortalization of HEK293 and natural integration of the human papilloma virus in the HeLa model.

**Fig. 3:**
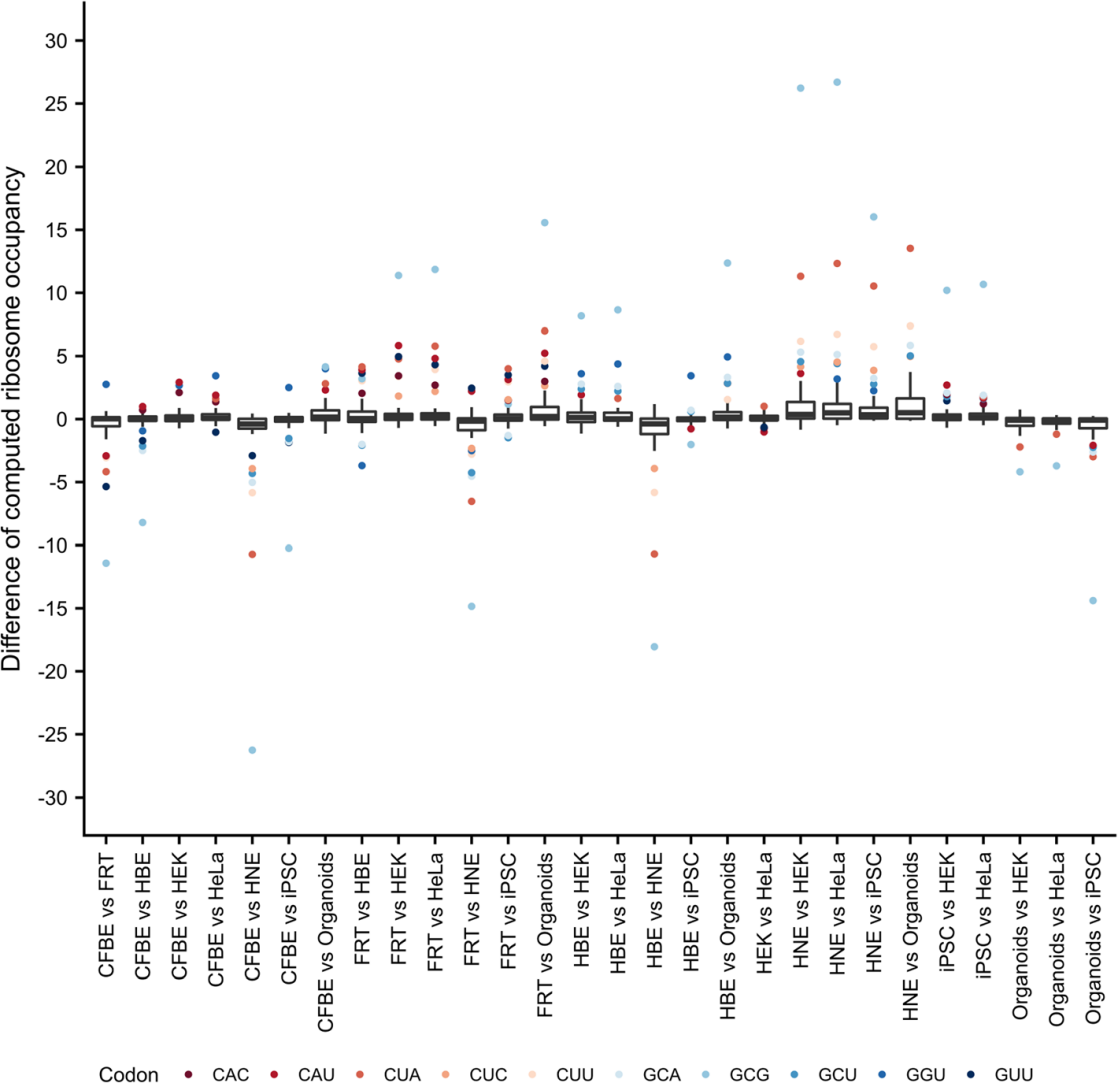
Ribosome occupancy at specific codons varies between models. Boxplots are shown describing pairwise differences in computed ribosome occupancy for each codon between two models. For iPSCs, only the zero time point was considered. Codons with differences in the upper and lower quantiles are designated with their identities. Codons found repeatedly in over ten combinations are color-coded

To further assess the influence of different tRNA concentrations on velocity of translation for each codon, tRNAs reading more than one codon were divided among cognate codons using the corresponding genomic codon frequencies. The variation of translational speed for some codons was very large (e.g. GCG, CUA, CAU), while some were equivalent regardless of the model being tested (e.g. GAC, GAU, AGG) (Additional file 1: Figure S3). Next, we calculated differences in tRNA fraction per codon in pairwise fashion. Codons falling in the upper and lower quantiles are marked (Fig. 3). Among the latter cohort, Ala-GCG was the most extreme (frequent) outlier and would be expected to have the strongest fluctuations in translational velocity among the tested models.

### Computing translation velocity

Codon frequencies, or codon adaptation index (CAI) [47], are often used for gauging translation speed as they can be easily inferred from genomic information and reflect core algorithms used to compute translation profile [48, 49]. The underlying assumption of these measures is that codon usage of highly expressed genes mirrors cellular tRNA pools. Although this correlation has been observed in prokaryotes and unicellular eukaryotes, it has not been reproduced in higher eukaryotes [22, 50]. An alternative metric that captures both codon frequency bias and tRNA abundance is the tRNA adaptation index (tAI), which applies copy number of tRNA genes and assumes correlation with tRNA abundance [51–53]. This approach also poses limitations based on discovery of tissue-specific variation in expression from tRNA genes, and consequently, in tRNA abundance among human tissues despite identical genetic information (i.e. tRNA copy number or genomic codon usage [46]). To capture differences in tRNA expression level in multicellular eukaryotes, we developed a RiboTempo algorithm that uses tRNA concentrations to compute translation speed for each codon [54, 55]. Codons pairing to low-abundance tRNAs represent slow codons, while those read by abundant tRNA species (Fig. 1) are rapidly translated codons. The average rate of translation along the entire transcript is computed by smoothing single codon values with a sliding window representing the size of mRNA covered by a translating ribosome [54].

### Predicting the impact of synonymous SNVs on translation rate

**Fig. 4:**
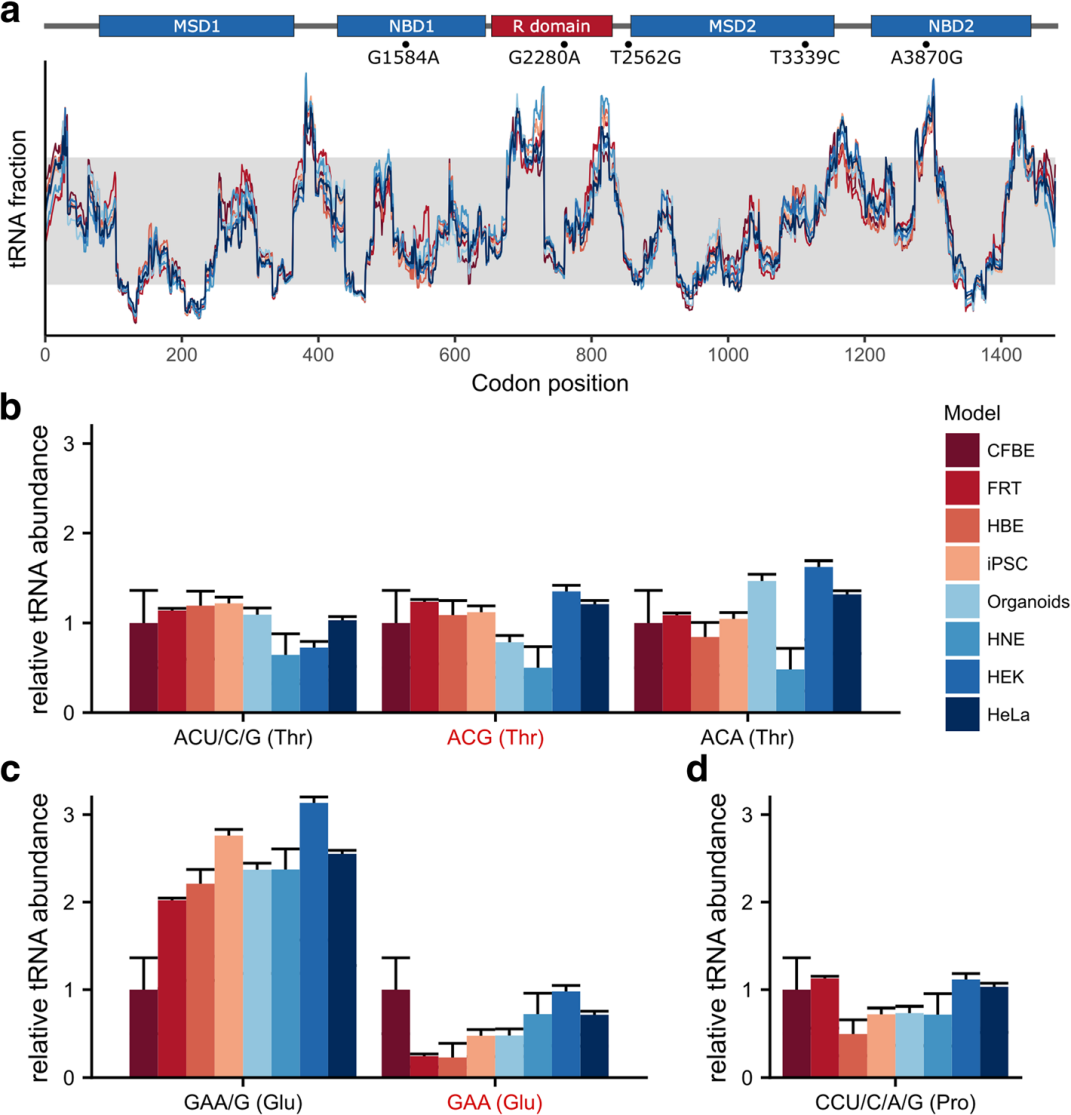
CFTR translation is predicted to occur via non-uniform velocity, with synonymous mutations locally governing the rate of synthesis. **a** Computed translation rate of CFTR for various tested cell or tissue models. The gray area marks the 10th–90th percentile to emphasize slow and rapidly translated regions. Single CFTR domains are designated as color-coded bars at the top of the sequence: membrane-spanning domains 1 and 2 (MSD1, MSD2) (blue); nucleotide-binding domains 1 and 2 (NBD1, NBD2) (blue); regulatory (R)-domain (red). sSNPs with prevalence higher than 1% in *CFTR* are marked with (●), and the nucleotide exchange at the corresponding position is designated using the scheme: wild-type nucleotide, position, mutated nucleotide. **b-d** tRNA concentration within the tRNA^Thr^ (**b**), tRNA^Glu^ (**c**) and tRNA^Pro^ (**d**) families compared to those present in CFBE41o^−^ cells. The synonymous SNP-induced codon is designated in red. For iPSCs, only the zero time point was reported in all panels. tRNA isoacceptors are depicted with their cognate codon and the corresponding amino acid

Within a single model, tRNAs varied in abundance by an order of magnitude, and certain synonymous codons exhibited several-fold differences (Fig. 2). Using tRNA fractions per codon (i.e., with abundance of each tRNA represented as a fraction of the total tRNAome for each cell or tissue), we computed translation profiles for the *CFTR* transcript based on a RiboTempo algorithm [54] and observed non-uniform velocity (Fig. 4a). In this representation, valleys correspond to slowly-translated regions and peaks to rapidly-translated segments. Overall, the *CFTR* translation profile was similar among models, although subtle differences were noted in specific regions (e.g. discrete positions in the R-domain and membrane-spanning domains (MSDs) 1 and 2 were translated more rapidly in some CF cell models, Fig. 4a). In two regions – between codons 500–570 in nucleotide-binding domain 1 (NBD1) and at the beginning of NBD2 – we noted alterations of the local speed of translation between models (Fig. 4a). Since translation kinetics tunes synthesis at critical nodes of the CFTR co-translational folding landscape [56], it is likely that local alterations in the speed of translation may impact biogenesis and yields of active protein in certain CF models.

Our earlier observations in CFBE41o^−^ cells indicate that the synonymous c.2562 T > G SNP (Fig. 4a) modifies local speed at Thr854 by introducing a codon (ACG) that pairs to a very low-abundance tRNA compared to the wild-type codon (ACT) with high-abundance tRNA [22]. Hence, we next compared concentrations of the tRNAs^Thr^ isoacceptor family members of each model to those present in CFBE41o^−^ (Fig. 4b). Since the concentration of both tRNAs^Thr^ pairing to ACU/C/G and ACG, respectively, is 1 in FRT, HBE, HeLa and iPSC (Fig. 3b), or equal to these in CFBE41o^−^, it would be expected that the c.2562 T > G SNP would have the same effect in these models. In organoids, tRNA^Thr^ pairing to ACG is much lower than in CFBE41o^−^ cells, and consequently, the predicted effect on translation velocity would be even stronger (Fig. 4b). In contrast, both tRNAs^Thr^ pairing to ACU/C/G and ACG codons are lower in HNE than CFBE41o^−^ cells. Between these two tRNAs, however, Thr-tRNA^CGU^ that reads ACG codon is diminished, such that the effect of the synonymous c.2562 T > G SNP would be still detected, although significantly less pronounced. In HEK293 cells, concentrations of both tRNAs pairing to wild-type ACT and mutant ACG codons deviate in a direction opposite from those in CFBE41o^−^ cells (Fig. 3b), which is in line with our earlier experimental observation that the synonymous c.2562 T > G SNP does not impact CFTR expression level and function in HEK293 [22]. It should be noted that the tRNA^Thr^AGU pairing to ACU/C/G codons has an A nucleotide at position 34 in the anticodon loop. An A34 at this position is often deaminated to inosine [57], which expands the coding capacity to pair with U/C/A (and likely G) at the third nucleotide of codons [58]. Yet, modifications of this sort in the human tRNA^Thr^ family are not known (Modomics - tRNA modification data base, http://modomics.genesilico.pl/), and it is therefore unclear whether Thr-tRNA^AGU^ would read an ACG codon. Furthermore, the presence of A34 is advantageous as a means to pair with fully degenerate synonymous codons by establishing a single tRNA that reads all four codons, except when tRNAs dedicated to some of the synonymous codons of interest are present [59], implying that Thr-tRNA^AGU^ may not pair to an ACG codon which has its own isoacceptor.

Similar to c.2562 T > G, two additional synonymous SNPs (c.1584G > A and c.3870A > G) reduced expression of mature CFTR protein in CFBE41o^−^ cells [22]. To determine whether these sSNPs alter local translation speed at each respective codon, we compared concentrations of their cognate tRNAs in different models. The synonymous c.1584G > A SNP, which is located in NBD1 (Fig. 4a), exchanges GAG to the GAA codon and is of considerable interest with regard to the concentration of the tRNA isoacceptor. This particular synonymous SNP would be expected to increase local speed of translation at the affected codon, with concentrations of cognate tRNAs that differ by 2-fold in CFBE41o^−^ cells (Fig. 2). In all other models, the concentration difference between tRNAs is substantial (e.g. the tRNA reading GAA/G is much higher and the tRNA pairing to GAA is much lower than in CFBE41o^−^ cells, except for organoids); thus, the effect of the G1584A synonymous SNP would be even stronger than observed in CFBE41o^−^ (Fig. 4c).

The synonymous c.3870A > G SNP (Fig. 4a) introduces changes at the Pro1290 codon, but modifications in local translation rate could not be computed due to high sequence similarity of the tRNA^Pro^ isoacceptors – which are not unambiguously distinguished by the arrays (Fig. 4d and Additional File, S1). Differences among the tRNAs^Pro^ family in these models suggest that the effect of the c.3870A > G synonymous SNP on translation velocity may differ, but the direction of speed alteration with the synonymous SNP at the affected codon cannot be predicted.

**Fig. 5:**
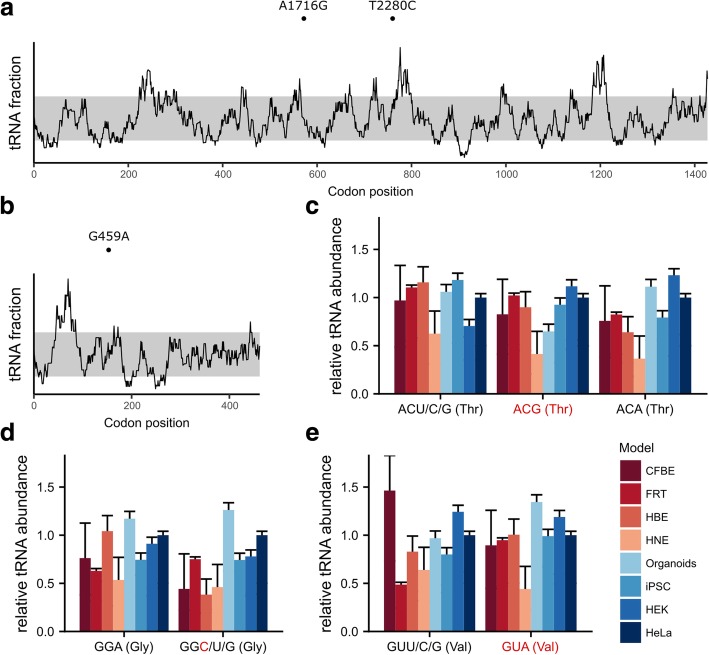
Predicted translation profiles for *ADAMTS13* (**a**) and blood coagulation factor IX (*F9*) (b) transcripts computed with RiboTempo using the tRNA concentration of HeLa cells. The gray area marks the 10th–90th percentile. The position of exemplified sSNVs is marked with (●) and designated using the scheme: wild-type nucleotide, position, mutated nucleotide. **c-e** tRNA concentration within the tRNA^Thr^ (**c**), tRNA^Gly^ (**d**) and tRNA^Val^ (**e**) families compared to those present in HeLa cells, which are set to 1. The synonymous SNP-induced codon is designated in red. For iPSCs, only the zero time point was reported in all panels. tRNA isoacceptors are depicted with their cognate codon and corresponding amino acid

We next applied the approach to two other transcripts, *ADAMTS13* and blood coagulation factor IX (*F9*), for which synonymous SNVs that change expression levels and/or conformation and function of the encoded protein have been experimentally identified [60, 61]. Similar to *CFTR* (Fig. 4a), *ADAMTS13* and *F9* transcripts are predicted to be translated with non-uniform velocity (Fig. 5a,b). Two synonymous mutations, c.1716A > G at a Thr codon and c.2280A > G at a Gly codon, have been shown to alter expression yields of *ADAMTS13* in HeLa cells [60]. The synonymous c.1716A > G SNV in *ADAMTS13* is reminiscent of c.2562 T > G in *CFTR,* since it exchanges a Thr encoding codon (ACA) pairing to a more abundant tRNA^Thr^ for a mutant ACG codon read by a low-abundance tRNA (Fig. 2 and data on the absolute tRNA concentration in HeLa published in [22]). This suggests that the synonymous c.1716A > G SNV inverts translation velocity at the Thr codon in *ADAMTS13*. In FRT, HBE, CFBE41o^−^ and iPSC, the concentration of both tRNAs^Thr^ pairing to ACA and ACG, respectively, is 1 or equal to those in HeLa (Fig. 5c). Thus, it would be expected that the c.1761A > G SNV would have the same effect in these models. On the other hand, in organoids, HNE and HEK293 cells, tRNAs^Thr^ pairing to ACG and ACA exhibit very different patterns of abundance compared to HeLa, and the effect of the c.1716A > G SNV on translational speed at the Thr codon in *ADAMTS13* might not be detected. The c.2280A > G SNV at a Gly codon in *ADAMTS13* exchanges GGA to GGG, both of which are read by tRNAs with fairly similar concentrations in HeLa (Fig. 2). Hence, the effect of this synonymous SNV might not be linked to the speed of codon translation. However, differences in the levels of both tRNAs^Gly^ in HBE cells may potentially have an effect on the Gly codon velocity (Fig. 5d).

In *F9*, the synonymous c.459G > A exchange at a Val codon is clinically reported to result in mild haemophilia B and diminished factor IX expression [61]. In HeLa cells, tRNA pairing to the cognate GUG codon has higher concentration (by ~ 50%) than tRNA reading the mutant GUA codon (Fig. 2 and data on the absolute tRNA concentration in HeLa published in [22]), suggesting an effect of this synonymous SNV on velocity of codon translation. The similarity in tRNA abundance between HeLa, CFBE41o^−^, HBE and iPSC indicates a similar phenotype in these cell models (Fig. 5e). In contrast, based on levels of tRNA expression, the effect should be reverted in FRT cells and organoids, and would not be detectable in HNE cells (Fig. 5e). Together, our results suggest that for synonymous SNVs known to alter protein folding and/or expression levels, tRNA concentration holds potential for gauging the effect of the synonymous SNV on local codon speed and programmed velocity of translation. By comparing tRNA abundance among model systems, cell lines in which the effect would persist could be selected.

## Discussion

Examples of tissue-specific effects on genetic variation [21–23] intimately link SNVs to the concentration of components that comprise translation machinery, and more precisely, abundance of ready-to-translate tRNA molecules [25, 26]. Our results demonstrate that tRNA households for different model systems used in disease-related research and drug discovery are globally similar, although key variations among models may strongly impact the effect of SNVs and consequently influence folding, maturation or gating of particular CFTR mutations. Synonymous SNVs have been extensively employed as a marker of neutral mutation rates and genomic evolution [62]. In the past decade, several studies have challenged this view and shown an effect of synonymous SNVs on protein structure and function, mRNA stability and processivity, as well as cell fitness, survival and adaptation (reviewed in [63–65]). Local changes in mRNA structure can be estimated using predictive algorithms (e.g. Vienna package of the RNAfold algorithm). In addition, alterations of splicing signals can be assessed with a recently developed algorithm that allows identification without relying on conserved coding features [66]. Our systematic evaluation of tRNAomes establishes that synonymous SNVs can also alter programmed translation patterns with tissue- and cell-specific consequences – including information that can be used to predict genotype-phenotype modifications due to synonymous SNVs.

Characterizing the phenotypic impact of SNVs is a central challenge in the field of functional genomics. Currently, computational algorithms are used to predict ramifications and relevance of both polymorphisms and disease-associated variants. Software and reference transcript sets used for annotating variants in this manner can have a large effect on the resulting annotation. Several attempts have been undertaken to introduce guidelines defining potential causality of SNVs and to establish gold standards by which to verify functionality [67–69]. Despite these efforts, false-positive calls concerning some variants are still commonly noted. Ultimate proof for a functional effect requires experimental evidence, which is limited by the paucity of global biochemical and/or functional analyses that evaluate entire proteomes. The approach described here holds potential for determining genotype-phenotype association for synonymous SNVs, which are among the most difficult to evaluate and verify experimentally. Rigorous quantification of tRNAomes provides a single measure of functional importance for each SNV and can be used as a criterion for determining candidacy with phenotypic impact.

## Conclusions

In summary, synonymous SNVs can alter local ribosomal speed at specific codons, since translation velocity is modulated by abundance of cognate tRNAs. Synonymous SNVs exhibiting large differences in concentration of cognate tRNAs reading at a mutant versus wild-type codon are expected to affect both protein biogenesis and function. Variable concentrations of tRNA isoacceptors in different cell models allow determination of SNVs most likely to be neutral in certain models. Hence, the suitability of a model system for a specific purpose – and in particular, for drug screening – should be selected based on the responsible SNV.

## Methods

### Analyzed samples and culture techniques

HeLa cells (American Type Culture Collection (ATCC) no. CRM-CCL-2), HEK 293 cells (ATCC no. CRL-1573) and immortalized CFBE41o^−^ cells (kind gifts of Karl Kunzelmann, University of Regensburg, Germany and Dieter Gruenert, University of California San Francisco, USA) were maintained in Dulbecco’s modified Eagle’s medium (DMEM; PAN Biotech) or Earle’s minimal essential medium (MEM; Biochrom), supplemented with 10% fetal calf serum (FCS; PAN Biotech) and 2 mM L-glutamine (Gibco). CF patient-derived primary HBE cells from lung biopsies (patient 1, ΔF508/ΔF508; patient 2, ΔF508/G551D) were kindly provided by Raymond Frizzell and Matthew Glover (University of Pittsburgh, Pittsburgh, USA). HBE cells were obtained and isolated following informed patient consent (HSTB, University Pittsburgh, IRB approval #0506140) from lung transplant recipients at the Human Airway Cell Core of the University of Pittsburgh, cultivated on transwell filters at 37 °C and 5% CO_2_, trypsinized, pelleted, stored in RNAlater (Ambion), and shipped on dry ice. Each patient-derived HBE and organoid sample was considered a single biological replicate.

Generation of parental FRT epithelia have been described previously [70]. Cells were cultured in F12 Ham Coon’s modified nutrient mixture (Sigma) supplemented with 2.68 g NaHCO_3_, 850 μL 2 N HCl (pH 7.3) and 5% FBS per liter.

Primary human nasal epithelia (HNE) were purchased from the Research Development Program (RDP) Experimental Models Core at Emory University. Cells were acquired by nasal curettage through an Emory IRB approved protocol (IRB#00,042,577) following consent obtained by the Cystic Fibrosis Biospecimen Repository (CFBR). HNE were harvested from non-CF (*CFTR*^*WT/WT*^) individuals – codes NL124 (male) and NL117 (female) – processed, then expanded using the F + Y reprogramming method on irradiated 3 T3 cell feeder layers as previously described [71, 72].

Rectal tissue specimens were obtained by means of a suction biopsy device from CF patients (homozygous F508del) as part of regular patient care, and from healthy controls, following approval of the study protocol by the Medical Ethical Committee of the Erasmus MC University Medical Center. The isolation of intestinal crypts from intestinal biopsies and organoid culture were performed as described in detail elsewhere [73, 74]. In brief, epithelial tissue sheets (crypts) were separated from the underlying tissue layers using a Ca^2+^-chelating solution. Isolated crypts were embedded in Matrigel (BD Bioscience) and incubated in Wnt3a-, EGF-, Noggin and R-Spo-containing cell culture medium (WENR) at 37 °C to promote organoid formation. Culture medium (WENR) was refreshed every 2–3 days and organoids subcultured weekly. For analysis, organoid fractions (equivalent to ~ 300,000 cells) were collected 7 days after seeding in ice-cold advanced DMEM/F12 (Invitrogen). Organoids were washed twice in advanced DMEM/F12 to remove remaining traces of Matrigel, then snap-frozen in liquid nitrogen. Samples were stored at − 80 °C until further processing.

Airway progenitor directed differentiation of iPSCs was performed as previously described [41, 42] with slight modifications of the protocol. Briefly, timing and density was optimized leading to 3-day incubation during a definitive endoderm (DE) step, followed by a 1:4 clump-based split (~ 50–200 cells). Cells were then grown in CFKBRa until Nkx2.1 specification peaked at day 13. Post Nkx2.1+ progenitor specification, culture was again divided using a 1:4 clump-based split (~ 500–1000 cells). Culturing was continued in CFK media until day 21, which was the end point for this experiment. Aliquots at day 0, 5, 10, 15 and 21 were flash frozen in liquid nitrogen, and stored at − 80 °C until further processing for total RNA isolation.

### tRNA microarrays

Total RNA from cells or organoids was isolated using the TRIzol-method according to manufacturer’s protocol (Ambion). RNA integrity was assessed by 10%-denaturing PAGE. To fully deacylate tRNAs, 5 μg of total RNA was incubated for 45 min at 37 °C in 100 mM Tris-HCl buffer (pH 9.0). Deacylated samples were purified by precipitation with ethanol and one volume 100 mM NaOAc (pH 4.8), supplemented with 100 mM NaCl and glycogen (20 mg/mL). For subsequent normalization of arrays, each sample was spiked with three or four in-vitro transcribed tRNAs (2 μM of each), which do not cross-hybridize with human tRNA.

Fluorescently labeled RNA:DNA hairpin oligonucleotides were ligated to deacylated tRNA samples using T4 DNA ligase (NEB) for 1 h at room temperature. HEK were used as comparison and labeled with Atto647 oligonucleotides, whereas other samples were typically labeled with Cy3-labeled oligonucleotides. Labeled tRNAs were extracted using phenol/chloroform/isomylalcohol (Roth) and precipitated with ethanol. Efficiency of ligation was analyzed by 10%-denaturing PAGE, and comparison of fluorescent signals to total tRNA was visualized by staining with SYBR gold (Invitrogen).

1–2 μg of labeled tRNAs from analyzed samples and HEK were simultaneously hybridized for 16 h at 60 °C on a microarray chip containing 24 technical replicates of each full-length tDNA. The detailed experimental protocol [44] is available at protocols.io [doi:dx.doi.org/10.17504/protocols.io.hetb3en].

Scanned microarray slides were analyzed using in-house Python scripts. Briefly, median of the ratio of Cy3/Atto647 signals was normalized to spiked samples with ratio set to one. Similarity for each isoacceptor between each pair of two biological replicates was assessed using variance analysis (Additional file 1: Figure S1), and overall similarity between replicates determined by two-sided Kolmogorov-Smirnov test. For individual models, the biological replicate exhibiting lowest internal variation over all tRNA probes was selected as a representative example and depicted with mean standard deviation for all probes. The latter was used to compute ribosome occupancy, and as input for the RiboTempo algorithm.

### Data analysis

Absolute tRNA concentration in HeLa [22] was used as a baseline set to convert tRNA isoacceptor abundancies from comparative microarrays into absolute units represented as a fraction of total tRNA (Fig. 1). For tRNA isoacceptors pairing to more than one codon, the fraction per codon was determined using the corresponding codon-usage index. Values for codons read by more than one tRNA were summed. The fractions of all tRNAs for one species were normalized to 100%.

Ribosome occupancy was computed as a reciprocal of the tRNA concentration for each sense codon [55]. The putative rate of translation along sequences was computed with RiboTempo (https://www.chemie.uni-hamburg.de/institute/bc/arbeitsgruppen/ignatova/tools-and-algorithms.html) using a harmonic mean function within a sliding window of 30-codon width.

### Data availability

tRNA microarray data for FRT, HBE, HNE, organoids and iPSCs are provided as a source file in Additional file 2. tRNA microarray data for CFBE41o^−^; HEK293 and HeLa cells, including sequences of tDNA probes used for the arrays, have been deposited with the Gene Expression Omnibus (GEO) under accession number GSE53991 [22].

## Additional file

Additional file 1: **Figure S1.** Biological replicates of the comparative tRNA microarrays are highly reproducible. a-e Data for HBE (a), organoids (b), CFBE41o- (c), FRT (d) and HNE from a male (NL124) or female (NL117) individual (e) are shown as fold-enrichment (gradient ruler at the bottom) of tRNAs compared with HEK293 cells. Global reproducibility between each two replicates was assessed by Kolmogorov-Smirnov test (for all arrays *p* ≥ 0.9, i.e. very similar). The reproducibility for each tRNA probe was assessed by variability analysis of each two replicates and is presented as coefficient of variance. tRNA isoacceptors are depicted with their anticodon and corresponding amino acid; Meti-CAU, initiator tRNAMet pairing to the AUG codon. **Figure S2.** Correlation of tRNA abundance between tested models. a Pairwise correlation of tRNA isoacceptor abundancies. For iPSCs, only the zero time point was considered. R, Pearson correlation coefficient. b Correlation of tRNA isoacceptor abundancies for iPCSs over the course of differentiation. Stem cells at days 5, 10, 15 and 21 (gradient ruler) were compared to non-differentiated cells at time zero. **Figure S3.** Standard deviations of computed ribosome occupancies per codon among models. In these calculations, only the zero time point was considered for iPSCs. (DOCX 1932 kb)

Additional file 2: Source data tRNA microarrays. (XLSX 18 kb)

## Data Availability

All data generated or analyzed during this study are included in the article and its additional files.

## Abbreviations

CF: Cystic fibrosis
CFTR: Cystic fibrosis transmembrane regulator
FRT: Fischer rat thyroid
HBE: Human bronchial epithelia
HNE: Human nasal epithelia
iPSC: Induced pluripotent stem cells
MSD: Membrane-spanning domains
NBD: Nucleotide-binding domain
SNV: Synonymous single nucleotide variant
