# Supplementary material for: Assessing cell-specific effects of genetic variations using tRNA microarrays
Source: BMC Genomics. 2019 Jul 16;20(Suppl 8):549. doi: 10.1186/s12864-019-5864-1 (PMC6632033; doi:10.1186/s12864-019-5864-1)
Supplement: Supplementary file 1 — Figure S1. Biological replicates of the comparative tRNA microarrays are highly reproducible. a-e Data for HBE (a), organoids (b), CFBE41o- (c), FRT (d) and HNE from a male (NL124) or female (NL117) individual (e) are shown as fold-enrichment (gradient ruler at the bottom) of tRNAs compared with HEK293 cells. Global reproducibility between each two replicates was assessed by Kolmogorov-Smirnov test (for all arrays p ≥ 0.9, i.e. very similar). The reproducibility for each tRNA probe was assessed by variability analysis of each two replicates and is presented as coefficient of variance. tRNA isoacceptors are depicted with their anticodon and corresponding amino acid; Meti-CAU, initiator tRNAMet pairing to the AUG codon. Figure S2. Correlation of tRNA abundance between tested models. a Pairwise correlation of tRNA isoacceptor abundancies. For iPSCs, only the zero time point was considered. R, Pearson correlation coefficient. b Correlation of tRNA isoacceptor abundancies for iPCSs over the course of differentiation. Stem cells at days 5, 10, 15 and 21 (gradient ruler) were compared to non-differentiated cells at time zero. Figure S3. Standard deviations of computed ribosome occupancies per codon among models. In these calculations, only the zero time point was considered for iPSCs. (DOCX 1932 kb) [file 12864_2019_5864_MOESM1_ESM.docx]

**ADDITIONAL FILE**

**Assessing cell-specific effects of genetic variation using tRNA microarrays**

Christine Polte, Daniel Wedemeyer, Kathryn E. Oliver, Johannes Wagner, Marcel J.C. Bijvelds, John Mahoney, Hugo R. de Jonge, Eric J. Sorscher, Zoya Ignatova


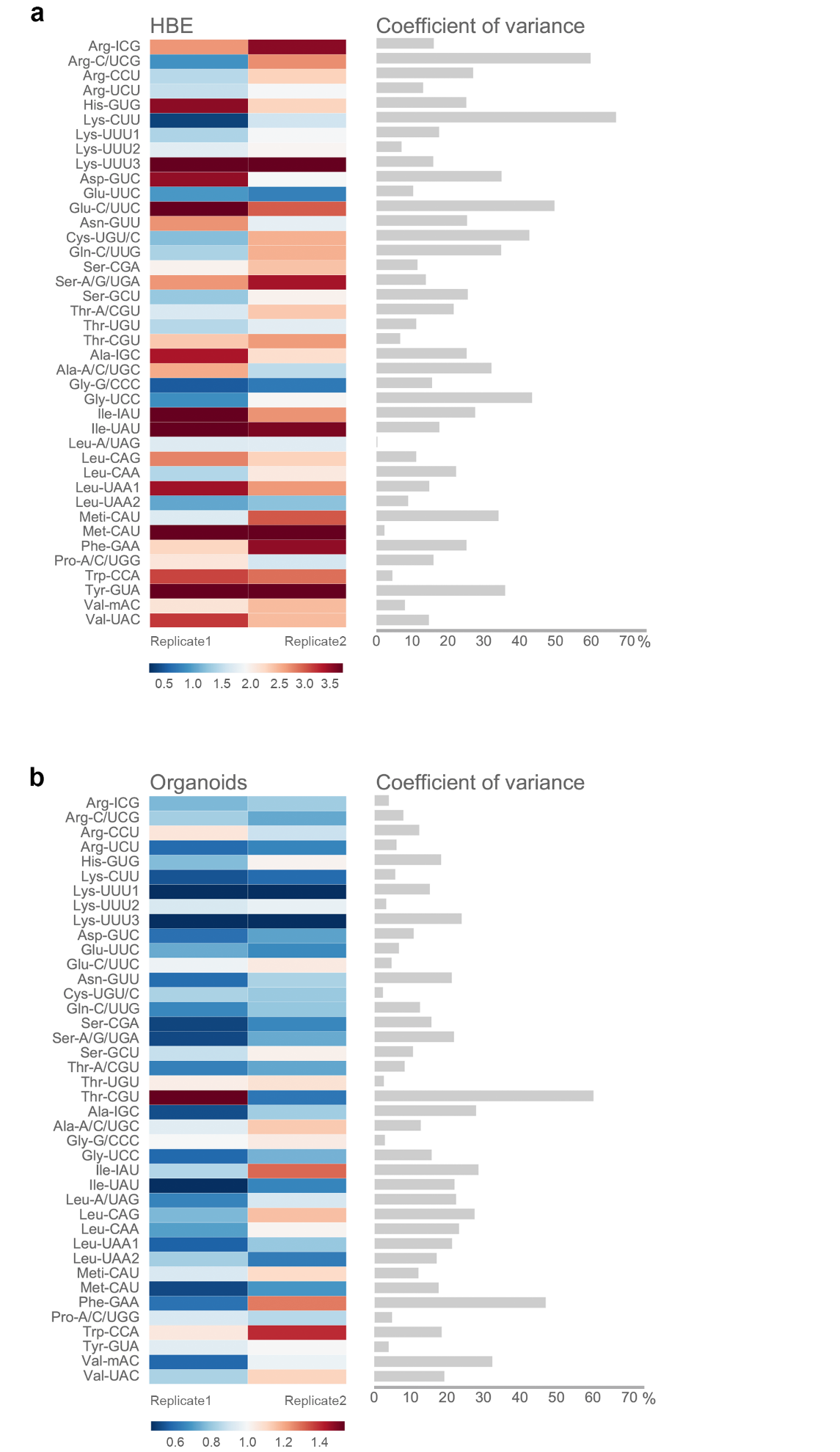


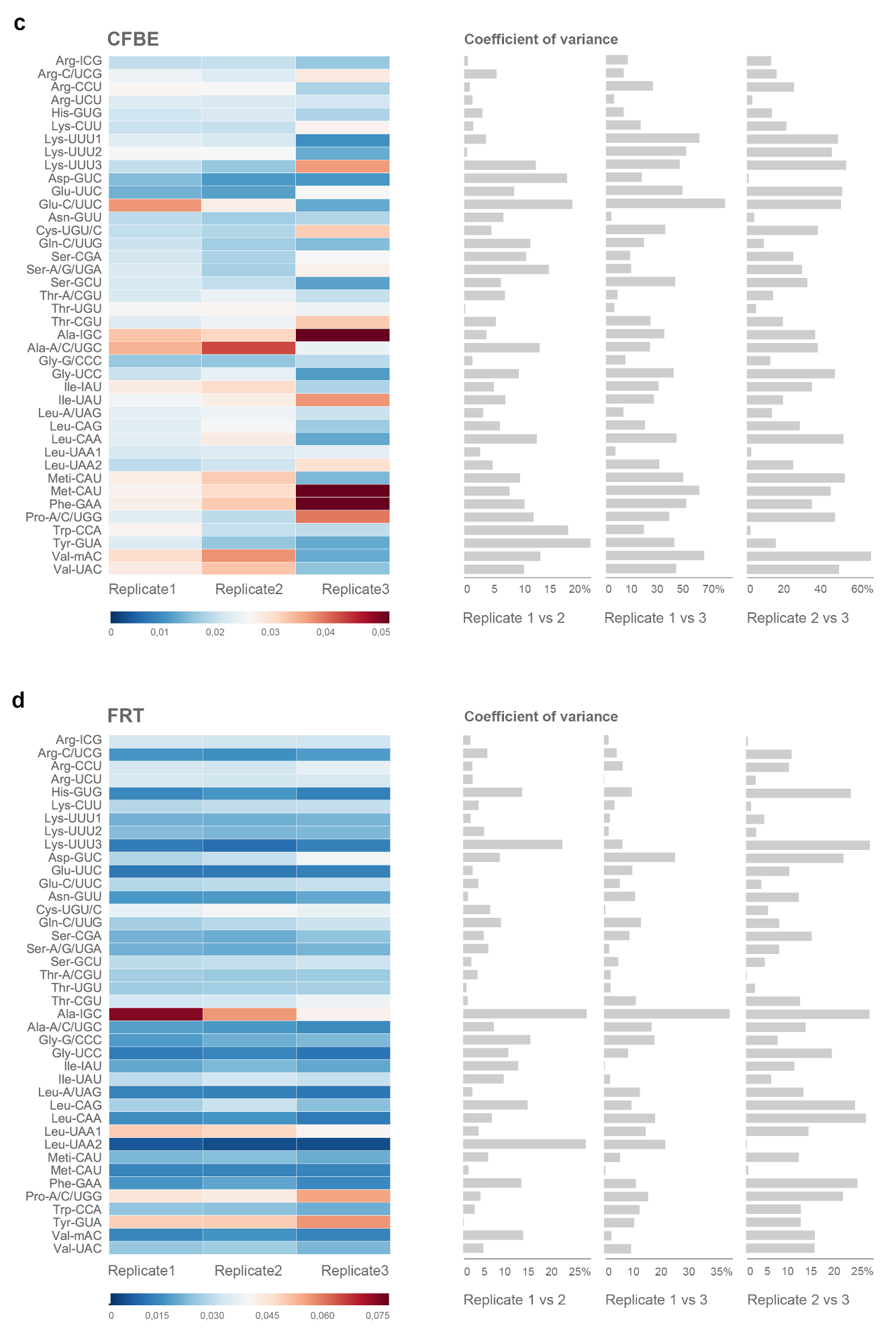


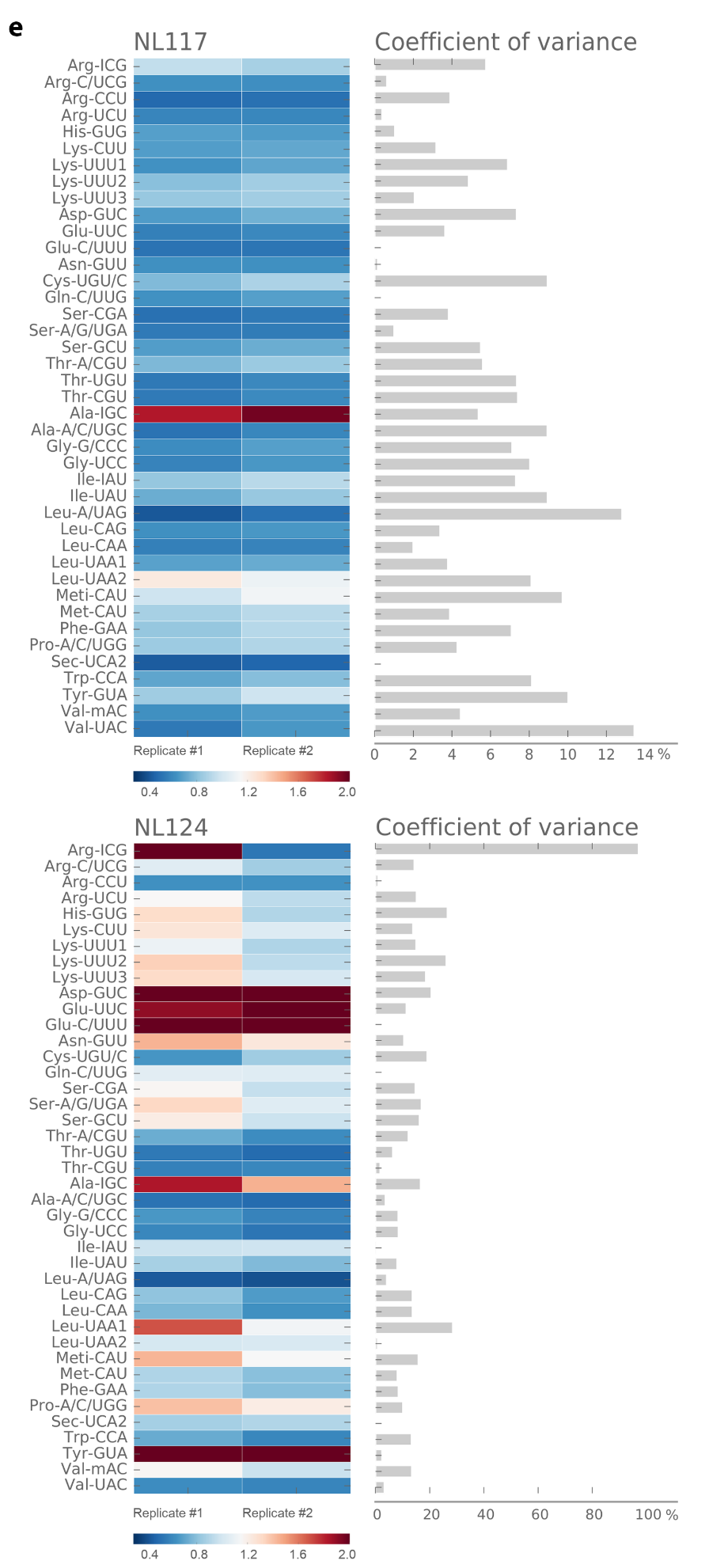


Fig. S1

Biological replicates of the comparative tRNA microarrays are highly reproducible. **a-e** Data for HBE (a), organoids (b), CFBE41o- (c), FRT (d) and HNE from a male (NL124) or female (NL117) individual (e) are shown as fold-enrichment (gradient ruler at the bottom) of tRNAs compared with HEK293 cells. Global reproducibility between each two replicates was assessed by Kolmogorov-Smirnov test (for all arrays p ≥ 0.9, i.e. very similar). The reproducibility for each tRNA probe was assessed by variability analysis of each two replicates and is presented as coefficient of variance. tRNA isoacceptors are depicted with their anticodon and corresponding amino acid; Meti-CAU, initiator tRNA^Met^ pairing to the AUG codon.


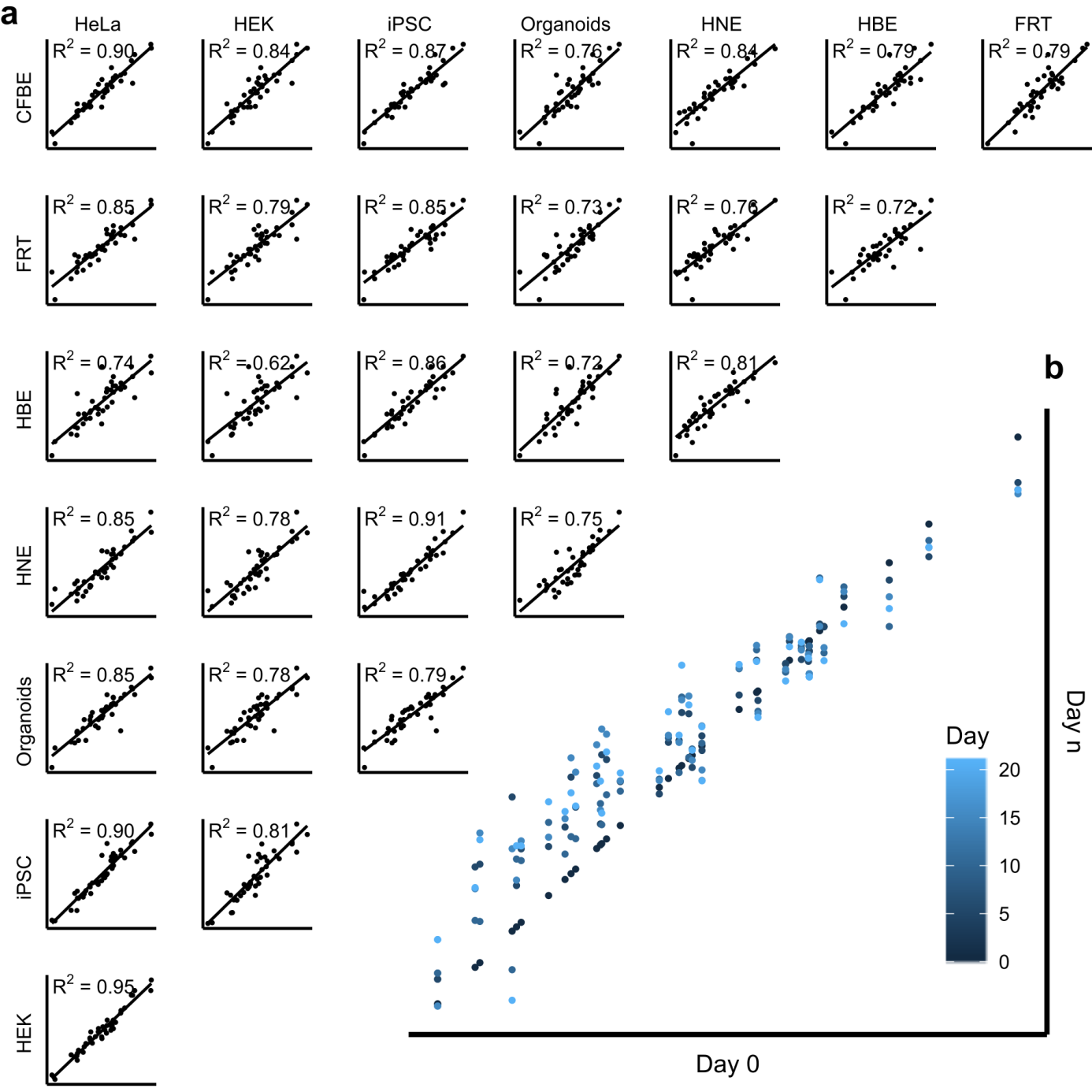


Fig. S2

Correlation of tRNA abundance between tested models. a Pairwise correlation of tRNA isoacceptor abundancies. For iPSCs, only the zero time point was considered. R, Pearson correlation coefficient. b Correlation of tRNA isoacceptor abundancies for iPCSs over the course of differentiation. Stem cells at days 5, 10, 15 and 21 (gradient ruler) were compared to non-differentiated cells at time zero.


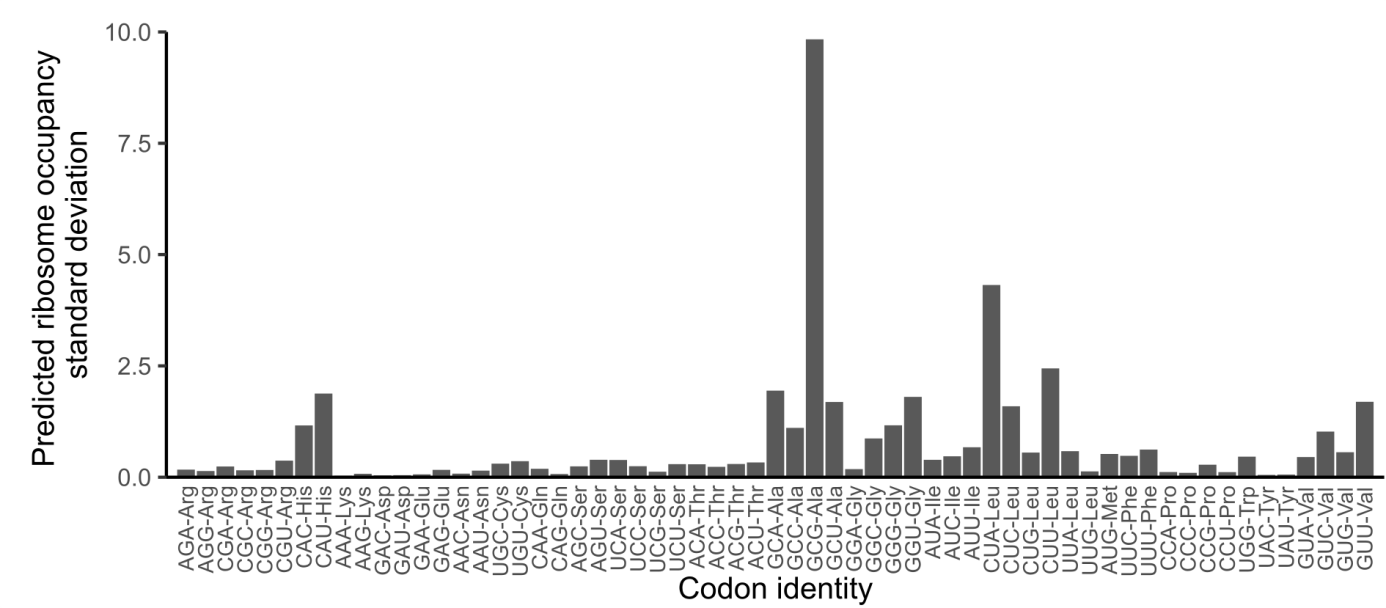


Fig. S3

Standard deviations of computed ribosome occupancies per codon among models. In these calculations, only the zero time point was considered for iPSCs.
